# Increased complexity of worker CHC profiles in *Apis dorsata* correlates with nesting ecology

**DOI:** 10.1371/journal.pone.0271745

**Published:** 2022-07-28

**Authors:** Fabienne Maihoff, Kyte Bohlke, Axel Brockmann, Thomas Schmitt

**Affiliations:** 1 Department of Animal Ecology and Tropical Biology, Biocentre, University of Würzburg, Würzburg, Germany; 2 National Centre for Biological Sciences, Tata Institute of Fundamental Research, Bangalore, Karnataka, India; University of Leipzig Faculty of Life Sciences: Universitat Leipzig Fakultat fur Lebenswissenschaften, GERMANY

## Abstract

Cuticular hydrocarbons (CHC) are known to serve as discrimination cues and will trigger defence behaviour in a plethora of eusocial insects. However, little is known how about nestmate recognition ability selects for CHC diversification. In this study we investigate differences in CHC composition of four major honey bee species with respect to the differences in their nesting behavior. In contrast to *A*. *mellifera*, *A*. *cerana* and *A*. *florea*, the giant honey bee *A*. *dorsata* prefers to build their nests in aggregations with very small spatial distances between nests, which increases the probability of intrusions. Thus, *A*. *dorsata* exhibits a particularly challenging nesting behavior which we hypothesize should be accompanied with an improved nestmate recognition system. Comparative analyses of the worker CHC profiles indicate that *A*. *dorsata* workers exhibit a unique and more complex CHC profile than the other three honey bee species. This increased complexity is likely based on a developmental process that retains the capability to synthesize methyl-branched hydrocarbons as adults. Furthermore, two sets of behavioral experiments provide evidence that *A*. *dorsata* shows an improved nestmate discrimination ability compared to the phylogenetically ancestral *A*. *florea*, which is also open-nesting but does not form nest aggregations. The results of our study suggest that ecological traits like nesting in aggregation might be able to drive CHC profile diversification even in closely related insect species.

## Introduction

**Fig 1:**
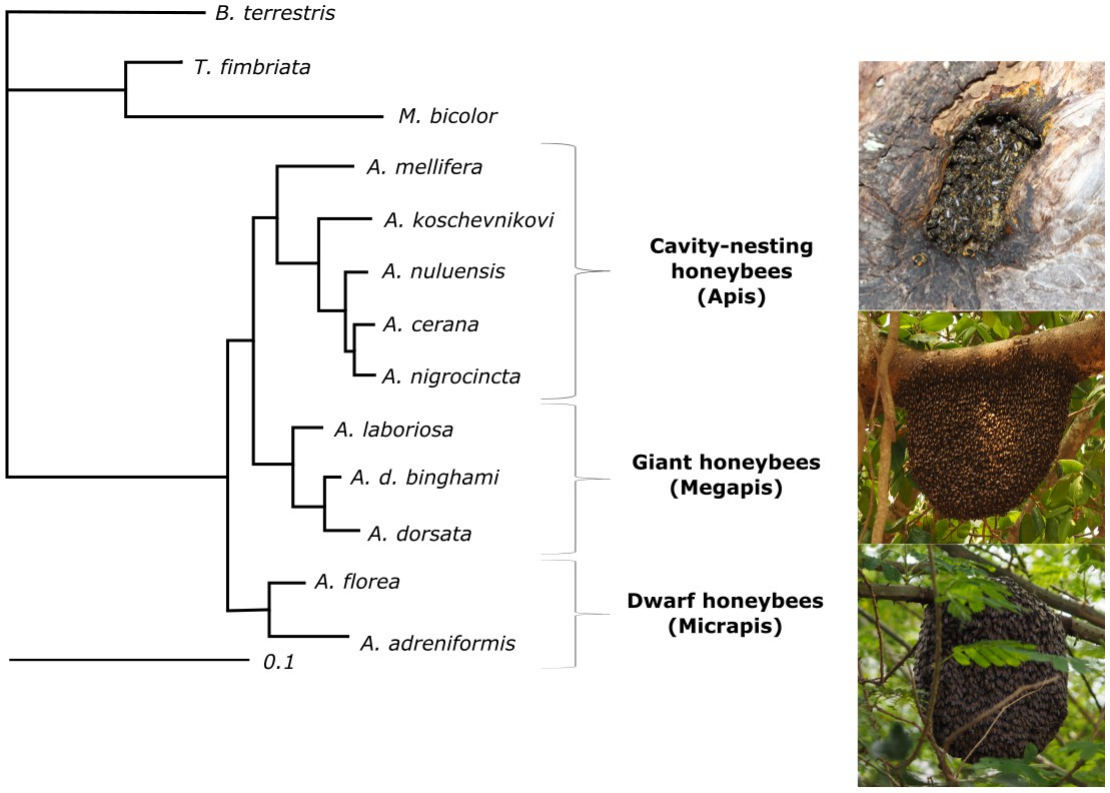
Apis phylogeny. Bayesian consensus tree, graph modified according to Raffiudin and Crozier [15]. Species of the genus *Apis* can be divided into cavity-nesting bees, giant honey bees and dwarf honey bees. They respectively nest in caves or have open nests, where the colony is either attached to the bottom of the substrate or surrounds the branch they are attached to. Bayesian consensus tree was derived from the data set omitting the third codon position of the cox2 sequence. The credibility values shown were derived from 2000 trees after burnin. *Trigona fimbriata*, *Melipona bicolor* and *Bombus terrestris* are included as outgroups.

Honey bees are a small group of closely related species showing distinct differences in their nesting behavior and ecology which affects the exposure to conspecific intruders (Fig 1) [1–4]. The dwarf honey bees (*A*. *florea* and *A*. *andreniformis*), which are the ancestral taxa, and the giant honey bees (*A*. *dorsata*, *A*. *laboriosa and A*. *d*. *binghami*) build open nests with single combs attached to the branches of bushes or trees and cliff overhangs. The workers of a colony form a flexible curtain, around the single comb which protects the brood but also allows free movement of the queen and workers between comb and curtain [2, 3, 5, 6]. The derived cavity-nesting species (species of the *A*. *cerana* group and *A*. *mellifera*) build their nests in tree trunks or other crevices with a small entrance hole. The different modes of nesting behavior require different strategies in colony defense [5]. While the cavity-nesting bees are protected by the cavity walls and a small entrance, the open-nesting bees are exposed to the environment, and the entire surface of the nest has to be protected against predators and intruders [7]. Besides these differences in nesting behavior, colonies of the giant honey bee species also tend to form nest aggregations. In *A*. *dorsata* it is common that 20 or even more colonies aggregate on one single tree or cliff site [8, 9]. Within an aggregation nests of individual colonies are in close vicinity to each other, sometimes even less than 1 m apart [10]. In contrast, nests of *A*. *florea* colonies are generally separated by larger distances of about 80–188 m [11]. Whether aggregation of nests in isolated tall trees are beneficial for *A*. *dorsata* by lowering the likelihood of being attacked by a predator, or aggregations are a consequence of the limited number of high quality nesting sites [7], the colonies within an aggregation are certainly at a higher risk of being intruded by drifting bees, robbers, and parasites. However, a very low frequency of drifted workers (0–6.25%) has been reported in aggregations [12, 13] suggesting an elaborate nestmate recognition system. Initially colonies respond to visual cues like irregular flight maneuvers of non-nestmates with shimmering behavior [14], whereas in close-range interactions after landing on the curtain olfactory cues like CHC profile differences are more likely used to identify the non-nestmate.

In addition to visual and acoustic cues that can be detected from a distance, chemical components of the insect cuticle such as cuticular hydrocarbons (CHCs) function as important close-range recognition cues particularly distinguishing from conspecifics which are similar in appearance and behavior [16–18]. CHCs have been shown to serve as cues for species and mate recognition as well as for communication [19–23]. CHCs comprise a diverse set of substances, which vary in chain length and in number and positions of double bonds (alkenes, alkadienes) and methyl-branches (methyl-, dimethyl-, trimethyl-branched alkanes). These long-chain hydrocarbons form the lipophilic layer of the cuticle. While long-chain hydrocarbon profiles are apparently species- or even colony-specific, they also show also a clear individual specificity [18, 24]. However, the functional role of single compounds or substance classes among the chemically diverse profiles is still under debate. Cuticular alkenes alone are sufficient to trigger aggression in *A*. *mellifera* [25] whereas in *Formica japonica* only the entire CHC profile trigger aggressive behavior [16]. In general, alkenes and methyl-branched alkanes, but not alkanes seem to play a crucial role in recognition processes [26–29].

In this study, we analysed and compared the variation in CHC profiles among major open- and cavity-nesting honey bee species. In particular, we were interested to explore whether the CHC composition of *A*. *dorsata* foragers differs specifically from all studied non-aggregating honey bee species (*A*. *florea*, *A*. *cerana*, and *A*. *mellifera*). Due to their nesting in aggregations, we hypothesized that the CHC profile of *A*. *dorsata* should contain more components relevant for recognition, e.g., alkenes and methylalkanes, which would increase the probability to distinguish non-nestmates from nestmates. Furthermore, we analysed the CHC profiles of worker pupae and compared them to those of the worker bees to investigate whether changes in the CHC profiles during the development may provide information that would help to reconstruct the evolutionary history of the CHC diversification in the genus *Apis*. This approach is based on theories of Darwin and the developmental constraints hypothesis [30–32], which assume a gradient of species divergence rates across ontogeny (embryonic < larval/pupal < adult).

In addition to the chemical analyses, we performed two types of behavioral assays, a dummy test and an experiment releasing nestmates and non-nestmates in front of a colony, to test differences in nestmate recognition abilities between colonies of the two open-nesting species *A*. *dorsata* and *A*. *florea*. We tested the hypothesis whether the colonies of both species can distinguish visually similar objects (dummies) using olfactory CHC cues, and whether *A*. *dorsata* worker show an improved discrimination between nestmates and non-nestmates than workers of *A*. *florea*.

## Material and methods

### Study region and time

All experiments were performed on honey bee colonies located on the National Centre for Biological Sciences (NCBS) and University of Agricultural Sciences UAS-GKVK campus in the North of Bangalore, India. *A*. *dorsata* and *A*. *florea* colonies and worker bees were obtained from bee removers that were called to remove colonies of *A*. *dorsata* from buildings and *A*. *florea* residential gardens. City residents are afraid of *A*. *dorsata* colonies nesting close to their apartments and it has become a quite common practice to eradicate *A*. *dorsata* colonies nesting at such buildings [33–35]. *A*. *florea* and *A*. *cerana* are not necessarily recognized as threats but requests to remove colonies from residential gardens have increased in recent years. Sample collections and behavioral assays were conducted during the dry seasons of 2015, 2017 and 2018. As resource availability may affect behavioral responses of bees, all experiments were conducted within the same season since high and equal food resource availability across experimental years can be assumed.

### Chemical analysis of cuticular hydrocarbon profiles

To explore possible phylogenetic changes in the CHC development among honey bee species, we analyzed the development of the CHC profiles from pupae to foragers. Six days old pupae were collected from brood combs kept in incubators. Following Groh and Rössler [36] pupal stages were identified with respect to color of the cuticle and the eyes. CHC profiles were extracted from flash-frozen pupae and foragers from two colonies per species. Each honey bee was extracted individually in n-hexane for 10 min. Extracts were evaporated in the fume hood, stored at -20°C and shipped to the University of Würzburg. Resuspended CHC extracts were analyzed with HP 6890 gas chromatograph coupled with a HP 5975 Mass Selective Detector (GC-MS, Agilent, Waldbronn, Germany): The GC (split/ splitless injector in splitless mode for 1 min, injected volume 1 μl at 300°C) was equipped with a DB-5 Fused Silica capillary column (30 m x 0.25 mm ID, *df* = 0.25 μm; J&W Scientific, Folsom, USA). Helium served as carrier gas at a constant flow of 1 ml/min. The following temperature program was used: Start temperature 60°C, temperature increase by 5°C per min up to 300°C, isotherm at 300°C for 10 min. The electron ionization mass spectra (EI-MS) were acquired at an ionization voltage of 70 eV (source temperature: 230°C). Chromatograms and mass spectra were recorded and quantified via integrated peak areas with the software HP Enhanced ChemStation G1701AA (version A.03.00; Hewlett Packard). CHC compounds were identified by the compound specific retention indices and their detected diagnostic ions [37].

We identified double-bound position in alkenes by DMDS-derivatization following Dunkelblum [38]. The GC settings were the same as described before, but oven temperature increased by 5°C per min up to 325°C and isotherm at 325°C for 10 min.

#### Comparative CHC analysis

We compared the relative abundances of compounds of pupae and worker bees of all four investigated *Apis* species. Only CHC compounds which contributed to at least 1% to the total profile were selected for the analysis. CHC profile similarity was assessed by Non-metric Multidimensional Scaling (NMDS) and agglomerative cluster analysis. Dissimilarities were calculated using Bray-Curtis distances. We revealed profile composition differences between bee species and developmental stages by performing permutational multivariate analysis of variance (MANOVA). All analyses were performed in R using the packages vegan [39] and pairwise Adonis [40]. Permutations were set on 10000 or 999 respectively.

Comparisons of the relative proportion of alkadienes, alkenes, alkanes, monomethyl alkanes and dimethyl alkanes were performed using Kruskal-Wallis rank sum test followed by Dunn test as post-hoc test (stats package, dunn.test package [41].

CHC diversity was calculated by the number of components contributing to the profiles (Component Richness) and calculating Shannon-Wiener-Indices. Peak areas including more than one compound were divided by the number of identified compounds. We calculated the Shannon-Wiener index per species based on the abundance values per component, averaged over all individuals of a species.

#### Nestmate odor recognition assay

We conducted dummy experiments to test a potential function of CHCs for nestmate recognition in *A*. *florea* and *A*. *dorsata*. The dummies consisted of pale beige Teflon tapes wrapped around pieces of a drinking straw (size adjusted to the tested species) impregnated with CHC extract from nestmates or non-nestmates respectively. The CHC extract was produced by immersing two bees in n-hexane for 10 min. The extract was concentrated to a volume of 150 ± 50μl and was applied drop by drop to the Teflon tape using a microliter glas pipet. Bee dummies were presented on a rod to the test colony for 6 seconds. To avoid that the dummy itself would cause a colony response by increasing a general alert [42] the dummy, attached to a thread and self-made fishing rod (made from a tree branch), was positioned on the surface of the bee curtain by a colony-specific adjusted speed and angle of movement [42]. The exact position on the curtain varied, but corresponded to the spots where nestmates returning in parallel to the experiment could be observed. Due to the restrictions in observation on the surface of the bee curtain, the behavior of workers was categorized as either high-aggressiveness, which was characterized by persisting stinging and biting, or low-aggressiveness/neutral which was characterized by brief gentle tactile and visual contact with the dummy (based on categorization in Harrison, Palmer & Rittschof [43].

Dummies treated with solvent (n-hexane) served as controls. The behavioral assay was performed blind in a random block design (each n = 40). Before the experiments, dummies without solvent or CHC treatment were presented ten times to the colonies to habituate the colony members successfully, thereby avoiding any responses elicited by presenting the dummy itself. During the experiments two focal colonies per species were used. To test whether colonies’ response differed between dummies with different odors and between species we modeled colony response (high-aggressiveness or low-aggressiveness) as a function of the interaction between species and dummy type using a GLMM with a binomial distribution. The test colony was modeled as random effect. We tested the interaction using a likelihood ratio test (ANOVA (fullmodel, model without interaction)). Data were analyzed using generalized linear mixed-effects models (GLMM) in the R language using the glmmTMB function [44]. Post-hoc tukey tests were performed with the emmeans function [45] (for summary statistics see S1 Table).

#### Worker releasing experiments

To test intruding attempts and intruder acceptance in nestmates and non-nestmates in *A*. *florea* and *A*. *dorsata* under natural condition, we collected workers from foreign colonies located at least 6 km away from the test colonies. Here, the distances exceed the expected gathering distances of both species, as resource availability in the landscape was very high at the time of our experiment, which is why at the same site and at the same phenological time even the larger *A*. *dorsata* forages only up to 3 km [46]. Workers were either collected directly from the bee curtain or from a feeder where bees were trained. Note that automated model selection based on the Akaike information criterion has shown that the models are not improved when this sampling type is integrated in one of the following analyses. Cohorts of around 30 foreign conspecific workers were marked and released in approximately 1.5 m from the test colony. Attempts to land on the bee curtain surface were observed. Bees were considered as accepted if they were able to move freely within the colony and stayed for at least 10 seconds without being expelled. As control we released cohorts of nestmates (originating from the test colony). Each of the four experimental combinations (2 species x nestmates/ non-nestmates) was repeated 10 to 13 times using a total of 4 test colonies per species (Table 1). To test whether foreign and nestmate workers differ in their tendency to enter the test colonies we modeled the number of intruding events (count data) as a function of the interaction between species and worker origin (nestmate vs non-nestmate) and the number of released individuals as an offset variable using GLMM with Conway-Maxwell-Poisson distribution, because it can handle both over- and underdispersion (Brooks et al. 2019). Colony was set as random effect. As we hypothesised that species would differ in their reaction towards nestmates and non-nestmates we tested the interaction using a likelihood ratio test (ANOVA (fullmodel, model without interaction)). We used the function emmeans to perform a post hoc tukey test to analyse pairwise differences between the four test categories (2 species x nestmates/non-nestmates). Note, that since we observed cohorts of released bees and not individual marked bees, the number of intruding attempts could exceed the total number of released bees. To test wether test colonies reacted differently to nestmates or foreign bees we modeled the number of observed accepting events (tolerance) as a function of the interaction between species and worker origin (non nestmates vs nestmates) and the total number of intruding attempts as an offset using a GLMM with Conway-Maxwell-Poisson distribution [47]. The test colony was modeled as random effect. Data were analyzed using generalized linear mixed-effects models (GLMM) in the R language using the glmmTMB function [44]. Post-hoc tukey tests were performed with the emmeans function [45] (for summary statistics see S1 Table).

**Table 1:** Sample size for intruder experiments.

	intruding attempts		intruding acceptance	
	n. of individuals	n. of cohorts	n. of individuals	n. of cohorts
*A*. *dorsta* nestmates	302	11	302	11
*A*. *dorsta* non-nestmates	370	13	288	10
*A*. *florea* nestmates	248	10	248	10
*A*. *florea* non-nestmates	357	12	332	11

Individual bees were released in cohorts: the table shows the total number of released bees and cohorts

## Results

### Cuticular hydrocarbon profile comparison

**Fig 2:**
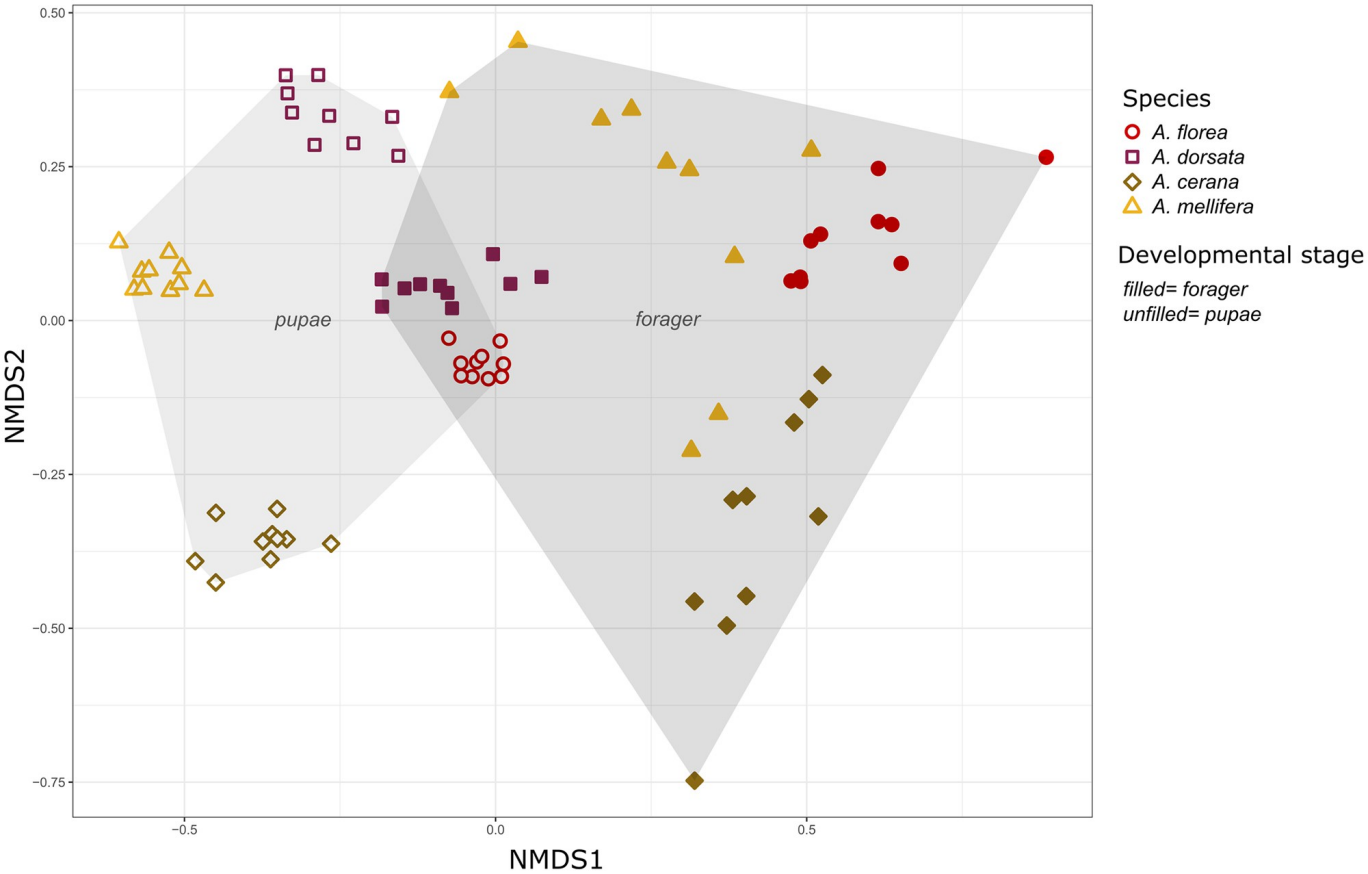
NMDS of CHC profiles. CHC profiles of pupae (unfilled) and foragers (filled) of the four honey bee species *A*. *florea* (circle), *A*. *dorsata* (square), *A*. *mellifera* (triangle) and *A*. *cerana* (diamond) displayed in a two-dimensional graph by non-metric multidimensional scaling (NMDS) of Bray-Curtis distances.

The CHC profiles of the four investigated species *A*. *florea*, *A*. *dorsata*, *A*. *cerana* and *A*. *mellifera* revealed species and developmental stage specific differences (see NMDS) (Fig 2, Tables 2 and 3).

**Table 2:** Permutational multivariate analysis of variance using distance matrices to test for an effect of species, developmental stage and their interaction on CHC profile composition.

Compared Treatments	DF	Sum of Squares	Mean Squares	F. Model	R^2^	p-value (based on 10000 permutations)
species	3	2.951	0.98367	16.709	0.40061	9.999e-05***
developmental stage	1	1.8852	1.88523	26.484	0.25592	9.999e-05***
Species: developmental stage	7	6.6105	0.94436	88.708	0.8973	9.999e-05***

Permutational Multivariate Analysis of Variance using Bray-Curtis distance matrices revealed an effect of species, developmental stage, and their interaction on CHC profile composition. Permutations were set to 10000. Levels of significance are indicated with stars (*p<0.05, **p<0.01, *** p<0.001).

**Table 3:** Pairwise permutational multivariate analysis of variance using distance matrices to reveal differences in the CHC profile composition between the species-developmental stage interactions.

Compared Treatments	Sum of Squares	F. Model	R^2^	p-value (based on 999 permutations)	p- adjusted (bonferroni)	
*A*. *florea*_p vs. *A*. *florea*_for	0.735	100.293	0.848	0.001	0.0028	**
*A*. *florea*_p vs. *A*. *dorsata*_p	0.648	107.046	0.863	0.001	0.0028	**
*A*. *florea*_p vs. *A*. *dorsata*_for	0.294	52.951	0.746	0.001	0.0028	**
A. florea_p vs. A. cerana_p	0.663	117.539	0.867	0.001	0.0028	**
*A*. *florea*_p vs. *A*. *cerana*_for	0.867	62.845	0.777	0.001	0.0028	**
*A*. *florea*_p vs. *A*. *mellifera*_p	0.661	162.836	0.900	0.001	0.0028	**
*A*. *florea*_p vs. *A*. *mellifera*_for	0.802	50.256	0.736	0.001	0.0028	**
*A*. *florea*_for vs. *A*. *dorsata*_p	1.186	145.735	0.896	0.001	0.0028	**
*A*. *florea*_for vs. *A*. *dorsata*_for	0.966	128.447	0.877	0.001	0.0028	**
*A*. *florea*_for vs. *A*. *cerana*_p	1.806	237.268	0.929	0.001	0.0028	**
*A*. *florea*_for vs. *A*. *cerana*_for	0.771	48.908	0.731	0.001	0.0028	**
*A*. *florea*_for vs. *A*. *mellifera*_p	1.891	313.538	0.946	0.001	0.0028	**
*A*. *florea*_for vs. *A*. *mellifera*_for	0.857	47.785	0.726	0.001	0.0028	**
*A*. *dorsata*_p vs. *A*. *dorsata*_for	0.563	89.921	0.841	0.001	0.0028	**
*A*. *dorsata*_p vs. *A*. *cerana*_p	0.787	123.863	0.879	0.001	0.0028	**
*A*. *dorsata*_p vs. *A*. *cerana*_for	1.362	90.819	0.842	0.001	0.0028	**
*A*. *dorsata*_p vs. *A*. *mellifera*_p	0.771	164.786	0.906	0.001	0.0028	**
*A*. *dorsata*_p vs. *A*. *mellifera*_for	0.888	51.378	0.751	0.001	0.0028	**
*A*. *dorsata*_for vs. *A*. *cerana*_p	0.829	142.121	0.888	0.001	0.0028	**
*A*. *dorsata*_for vs. *A*. *cerana*_for	1.038	74.183	0.805	0.001	0.0028	**
*A*. *dorsata*_for vs. *A*. *mellifera*_p	0.615	144.555	0.889	0.001	0.0028	**
*A*. *dorsata*_for vs. *A*. *mellifera*_for	0.689	42.642	0.703	0.001	0.0028	**
*A*. *cerana*_p vs. *A*. *cerana*_for	1.227	87.126	0.829	0.001	0.0028	**
*A*. *cerana*_p vs. *A*. *mellifera*_p	1.018	234.190	0.929	0.001	0.0028	**
*A*. *cerana*_p vs. *A*. *mellifera*_for	1.244	76.583	0.810	0.001	0.0028	**
*A*. *cerana*_for vs. *A*. *mellifera*_p	1.530	122.298	0.872	0.001	0.0028	**
*A*. *cerana*_for vs. *A*. *mellifera*_for	0.577	23.654	0.568	0.001	0.0028	**
*A*. *mellifera*_p vs. *A*. *mellifera*_for	1.135	77.356	0.811	0.001	0.0028	**

Test results for developmental differences (pupae (p) vs. forager stage (for)) within a species and species differences within a developmental stage are presented. Levels of significance are indicated with starts (*p<0.05, **p<0.01). Note, that due to 999 permutations the lowest possible achieved p value equal 0.001

**Fig 3:**
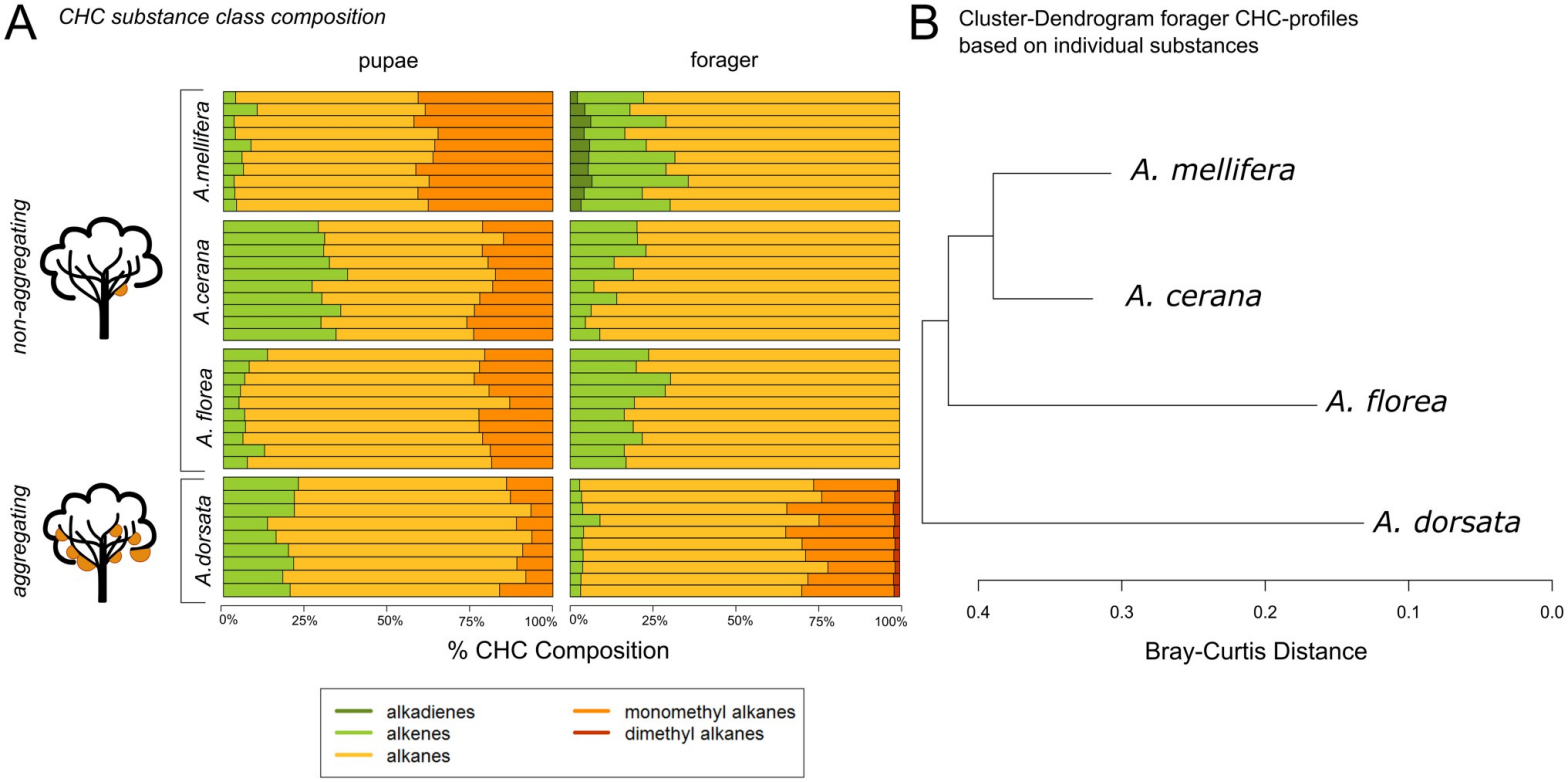
Comparison of hydrocarbon substance classes. (A) Relative proportions of hydrocarbon substance classes (alkadienes: dark green, alkenes: light green, alkanes: yellow, monomethyl alkanes: orange and dimethyl alkanes: red) in the pupal and forager stages in *A*. *mellifera*, *A*. *cerana*, *A*. *florea* and *A*. *dorsata* are presented. Each bar refers to one individual per species. (B): Cluster dendrogram of the forager CHC profile based on individual substances is shown.

We identified five substance classes: alkadienes, alkenes, alkanes, monomethyl alkanes and dimethyl alkanes, which differ in their relative abundance in the CHC profiles of the studied honey bee species (Fig 3(A), Table 4). In the pupal stage workers of all species have qualitatively similar profiles. All pupal profiles consist of alkenes, alkanes and monomethyl alkanes, but the relative compositions of the substance classes differ among the species. The pupal profiles of *A*. *florea* and *A*. *dorsata* are more similar to each other compared to *A*. *mellifera* and *A*. *cerana*. The pupal profile of *A*. *mellifera* is characterized by a relatively high proportion of monomethyl alkanes. In contrast, the pupal profile of *A*. *cerana* shows a high proportion of alkenes compared to the other species.

**Table 4:** Differences in mean (± SD) relative abundance of substance classes in the CHC profile of *A*. *mellifera*, *A*. *cerana*, *A*. *florea* and *A*. *dorsata* for pupae and foragers.

Substance class	species/ developmental stage	Pupae	Forager	Difference Kruskal-Wallis	Post-hoc Dunn’s Test
**Alkadienes**	*A*. *florea (f)*	0	0	—	—
	*A*. *dorsata (d)*	0	0	—	—
	*A*. *cerana (c)*	0	0	—	—
	*A*. *mellifera (m)*	0	0.05 ± 0.01	χ^2^(1) = 16.309, p = 5.381e-05***	—
	Pupae	—	—	—	—
	Forager	—	—	χ^2^(1) = 37.956, p = 2.887e-08***	f^a^, d^a^, c^a^, m^b^
**Alkenes**	*A*. *florea (f)*	0.08 ± 0.03	0.21 ± 0.05	χ^2^(1) = 14.286, p = 1.571e-04***	—
	*A*. *dorsata (d)*	0.19 ± 0.03	0.04 ± 0.02	χ^2^(1) = 13.500, p = 2.386 e-04***	—
	*A*. *cerana (c)*	0.32 ± 0.03	0.14 ± 0.07	χ^2^(1) = 14.286, p = 1.571e-04***	—
	*A*. *mellifera (m)*	0.05 ± 0.02	0.21 ± 0.06	χ^2^(1) = 14.286, p = 1.571e-04***	—
	Pupae	—	—	χ^2^(3) = 33.082, p = 3.094e-07***	f^bc^, d^ac^, c^a^, m^b^
	Forager	—	—	χ^2^(3) = 24.322, p = 2.14e-05***	f^b^, d^a^, c^b^, m^b^
**Alkanes**	*A*. *florea (f)*	0.72 ± 0.05	0.79 ± 0.05	χ^2^(1) = 6.223, p = 0.01261*	—
	*A*. *dorsata (d)*	0.70 ± 0.05	0.68 ± 0.04	χ^2^(1) = 0.96, p = 0.3272	—
	*A*. *cerana (c)*	0.48 ± 0.05	0.86 ± 0.07	χ^2^(1) = 14.286, p = 1.571e-04***	—
	*A*. *mellifera (m)*	0.55 ± 0.03	0.74 ± 0.06	χ^2^(1) = 14.286, p = 1.571e-04***	—
	Pupae	—	—	χ^2^(3) = 31.695, p = 6.068e-07***	f^b^, d^b^, c^a^, m^a^
	Forager	—	—	χ^2^(3) = 23.235, p = 3.608e-05***	f^ac^, d^b^, c^a^, m^bc^
**Monomethyl alkanes**	*A*. *florea (f)*	0.20 ± 0.03	0	χ^2^(1) = 16.309, p = 5.381e-05***	—
	*A*. *dorsata (d)*	0.11 ± 0.03	0.27 ± 0.04	χ^2^(1) = 13.500, p = 2.386e-04***	—
	*A*. *cerana (c)*	0.21 ± 0.03	0	χ^2^(1) = 16.309, p = 5.381e-05***	—
	*A*. *mellifera (m)*	0.39 ± 0.03	0	χ^2^(1) = 16.309, p = 5.381e-05***	—
	Pupae	—	—	χ^2^(3) = 31.162, p = 7.86e-07***	f^ab^, d^b^, c^a^, m^c^
	Forager	—	—	χ^2^(3) = 37.956, p = 2.887e-08***	f^a^, d^b^, c^a^, m^a^
**Dimethyl alkanes**	*A*. *florea (f)*	0	0	—	—
	*A*. *dorsata (d)*	0	0.02 ± 0.004	χ^2^(1) = 15.088, p = 1.026e-04***	—
	*A*. *cerana (c)*	0	0	—	—
	*A*. *mellifera (m)*	0	0	—	—
	Pupae	—	—	—	—
	Forager	—	—	χ^2^(3) = 37.956, p = 2.887e-08***	f^a^, d^b^, c^a^, m^a^

Comparisons of the relative proportion of substance classes were performed using Dunn test as post-hoc test after Kruskal-Wallis rank sum test. Mean relative proportion of substance class and standard error is presented for pupae and forager stage of each species. Kruskal-Wallis test results refer to developmental stage difference within a species or respectively overall species difference within a developmental stage. Degrees of freedom are presented in parenthesis. Levels of significance are indicated with stars (*p<0.05, **p<0.01, *** p<0.001). Pairwise species differences within developmental stage (Dunn test) are indicated with superscript letters.

In contrast to the pupal profiles, the forager profiles show strong qualitative differences in the composition of the substance classes among species. Alkenes and alkanes are represented in all species. However, monomethyl alkanes and dimethyl alkanes occur only in *A*. *dorsata* forager profiles whereas small amounts of alkadienes occur exclusively in *A*. *mellifera* forager profiles. Overall, *A*. *florea*, *A*. *mellifera* and *A*. *cerana* forager profiles are more similar with respect to the proportions of alkanes and alkenes compared to the profiles of *A*. *dorsata*.

There is a shift from relatively high to low proportions of monomethyl alkanes from pupae to forager stage in all species except *A*. *dorsata*. In contrast, *A*. *dorsata* increases the proportion of monomethyl alkanes during development from pupae to forager and adds dimethyl alkanes to the profile (Fig 3, Table 4).

A cluster dendrogram analysis of CHC profiles of foragers of all species based on the comparison of single components revealed that *A*. *mellifera* and *A*. *cerana* have the most similar CHC profiles. Most interestingly, the foragers of the open-nesting and aggregating *A*. *dorsata* have the most distinct CHC profile (Fig 3(B)), separating it from all the other studied species. Consequently, the cluster dendrogram based on CHC profiles deviates from the well-established phylogeny of honey bees (Raffiudin & Crozier, 2007 see Fig 1). In addition, a NMDS of the CHC profiles of 28 workers from three different colonies of *A*. *dorsata* and of 31 workers from three different colonies of *A*. *florea*, show a separation of each colony with some overlap (S1 File).

**Fig 4:**
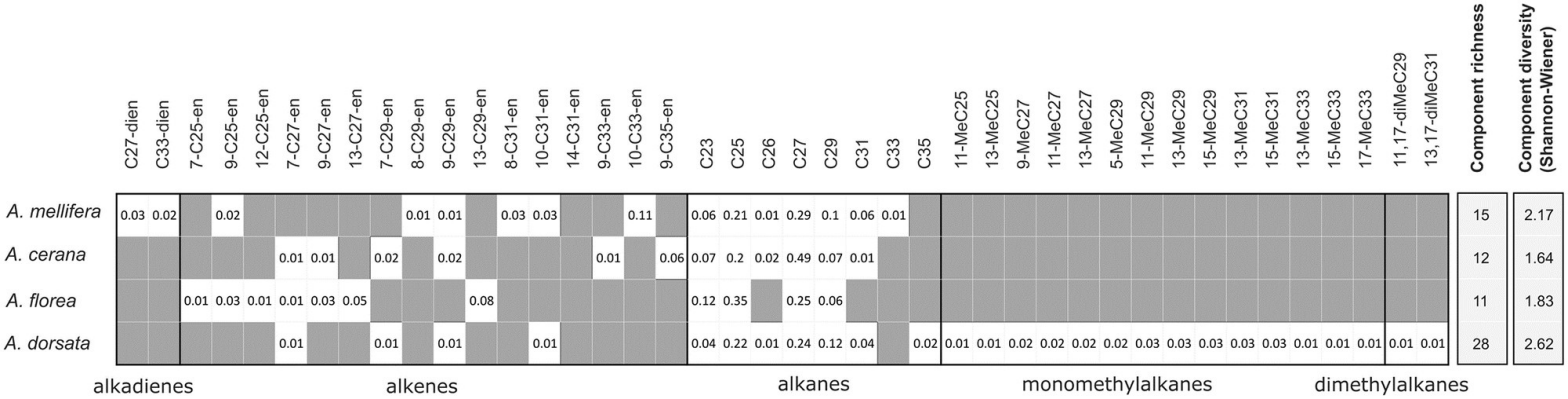
Survey on the components summarizing species-relevant factors. Honey bee species differ in their mean component richness and diversity (calculated according to Shannon-Wiener-Index). Additionally, abundances of single component are presented. The mean proportion of each component per species is given.

Averaged CHC profile diversity per species was estimated in forager profiles by calculating (1) the number of components contributing to the profile (component richness) and (2) the Shannon-Wiener Index, which accounts for the abundances of components. With regard to the total CHC profiles, *A*. *dorsata* has the highest component richness (28 components), which was around twice as high as in the other species. *A*. *mellifera* (15 components) had a higher richness than *A*. *cerana* (12 components) and *A*. *florea* (11 components), but differences between the three latter species are small. Considering Shannon-Wiener diversity, *A*. *dorsata* has the most diverse profile (2.62) followed by *A*. *mellifera* (2.17), *A*. *florea* (1.83) and *A*. *cerana* (1.64) (Fig 4).

### Behavioral approach: Cuticular hydrocarbons as nestmate recognition cues

**Fig 5:**
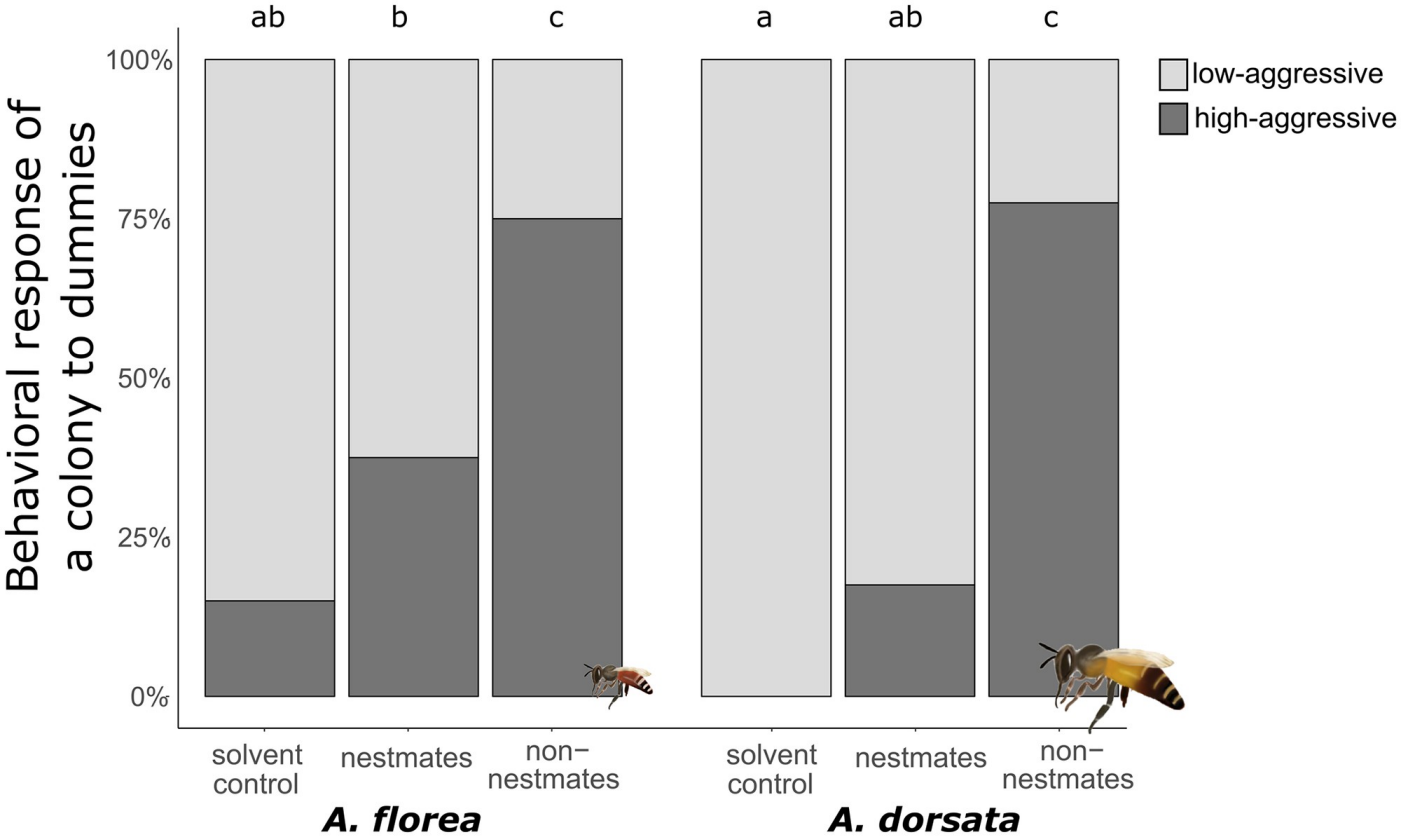
The behavioral response of a colony to odor dummy. The behavioral response of a colony (high-aggressive: dark grey: low-aggressive: light grey) to odor dummies is shown. Dummies applied with solvent served as control. Letters are indicating significant differences in the proportion of high-level aggressive and low-level aggressive colony responses achieved via post-hoc Tukey test. For all tests related to species x dummy type, 40 individual dummies were tested.

By exposing bee dummies covered with CHC extracts from nestmates or non-nestmates in front of colonies, we tested whether CHCs alone are sufficient to trigger differential response of *A*. *florea* and *A*. *dorsata* colonies towards nestmates and non-nestmates. The three dummy types (nestmate, non-nestmate and solvent control) elicited different degrees of high-level aggressive responses (factor dummy type: χ^2^ (2) = 89.84, p<0.001). In addition, the effect of the dummy did not significantly differ the behavior in both species (interaction: χ^2^ (2) = 4.5, p = 0.105) (Fig 5). Colony aggresiveness was lowest towards solvent control in both species, although *A*. *florea* workers showed an aggressive behavior against the solvent control dummy in a few cases. Colonies showed a significantly higher proportion of high-level aggressive responses towards the dummies with the non-nestmate CHCs than those with the nestmate CHCs. While in *A*. *florea* high-level aggressive behavior towards non-nestmates odor dummies was shown twice as often as towards nestmate odor dummies (75% vs 37.5%), in *A*. *dorsata* high-level aggressive responses towards the dummies with non-nestmate was more than four times higher (77.5% vs 17.5%).

### Behavioral approach: Intruder acceptance

**Fig 6:**
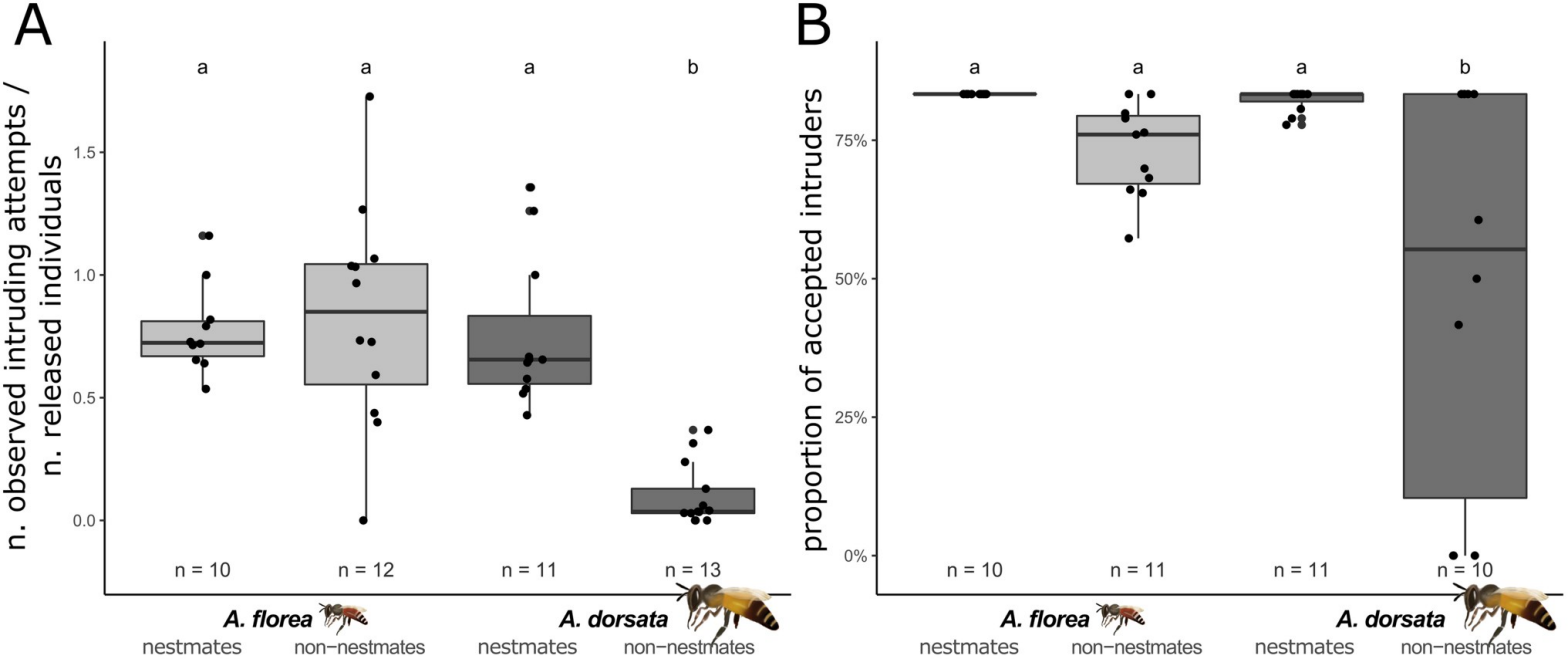
Intruding attempts and acceptance. (A) Differences in the proportion of intruding attempts (measured as the proportion of observed intruding individuals to released individuals) between nestmates and non-nestmates in *A*. *florea* and *A*. *dorsata* are presented. (B) Differences in the proportion of accepted individuals between released nestmates and non-nestmates and between species (*A*. *florea* and *A*. *dorsata*) are shown. Letters indicate differences in the proportion of accepted individuals achieved via post-hoc test (Tukey). The number of observed cohorts is indicated with n.

By releasing workers in front of conspecific colonies we determined the willingness of workers to enter their own or a foreign colony as well as the responses of the colonies towards the entering nestmates and non-nestmates. *Apis florea* and *A*. *dorsata* workers differed in their attempts to enter conspecific colonies (Glmm: χ^2^ (1) = 30.763, p < 0.001) (Fig 6(A)). In *A*. *florea*, it was observed that both colony members and foreign workers entered the test colony, with no significant differences in their behaviour when in contact with the colony (0.83 vs 0.78, Tukey: p = 0.96), while in *A*. *dorsata* foreign workers were observed to enter the test colony less frequently than nestmates (0.1 vs. 0.75, Tukey: p < 0.001).

Correspondingly, colonies of *A*. *florea* and *A*. *dorsata* differed in their response towards conspecific intruders (Glmm: χ^2^ (1) = 10.427, p < 0.01) (Fig 6(B)). Acceptance of non-nestmates (88%) and nestmates (100%) did not significantly differ in *A*. *florea* colonies (Tukey, p = 0.07), whereas *A*. *dorsata* colonies showed a significantly lower acceptance rate towards non-nestmates (58%) compared to nestmates (99%) (Tukey: p < 0.001).

## Discussion

The results of our study indicate that *A*. *dorsata* workers exhibit a more complex and qualitatively more diverse CHC profile than foragers of *A*. *florea*, *A*. *cerana*, and *A*. *mellifera*. This mirrors the nesting ecology of the studied species with *A*. *dorsata* beeing the only species that nests in aggregations, while all other species do not nest in such close proximity. In addition, *A*. *dorsata* workers avoid entering foreign colonies more often and *A*. *dorsata* guards recognize and repel intruders to a higher degree than *A*. *florea*. The results of our behavioral experiments indicate that colonies of *A*. *dorsata*, are better at maintaining their social integrity than colonies of *A*. *florea*. The correlation between a more complex CHC profile and more selective behavioral response toward non-nestmates suggests that *A*. *dorsata* might have evolved a more fine-tuned nestmate recognition system in parallel with the evolution of building their nests in close aggregations.

The variation of CHC profiles among all analysed honey bee species is driven by differences in the occurrence of alkenes, alkadienes, monomethyl alkanes and dimethyl alkanes. All these components have been demonstrated to play major roles in nestmate recognition, as insects are able to detect and differentiate CHCs with different number and positions of double bonds and methyl-branches [26, 29]. *A*. *dorsata* is the only honey bee species exhibiting methyl-branched alkanes (mono- and dimethyl alkanes) in the forager profile with a proportion of about 25% of the amount of CHCs. Thus, we propose that methyl-branched alkanes found in the forager profile likely play a functional role for the fine-tuned recognition system in *A*. *dorsata*. In contrast, alkanes, which are present in the forager profiles of all species, do not provide cues as good as unsaturated or methyl-branched hydrocarbons due to the lack of steric complexity [25, 26, 48, 49].

Interestingly, we found complex CHC profiles with a large proportion of methyl-branched alkanes on the pupae of all four investigated species of honey bees. Indeed, we show that all honey bee species produce alkanes, methyl-branched alkanes and unsaturated hydrocarbons at some point during their development. However, *A*. *mellifera*, *A*. *cerana* and *A*. *florea* reduce the abundance of methyl-branched alkanes when they develop into foragers. Genes encoding enzymes for monomethyl alkane biosynthesis might be downregulated or silenced at some point in the development. Only *A*. *dorsata* increases the proportion of methyl-branched alkanes during the development from the pupal to the adult stage. Thus, the CHC profile of *A*. *dorsata* foragers is more similar to the pupal stage of all four studied honey bee species than the forager profiles of *A*. *mellifera*, *A*. *cerana* and *A*. *florea*. Interestingly, the divergence pattern of the CHCs mirror the molecular phylogenetic tree except for the position of *A*. *dorsata* [3, 4, 15, 50]. This finding suggests that the CHC profiles in *A*. *dorsata* with a high content of methyl-branched alkanes are subject to selection. In contrast, the diversification of the CHC profiles in the other *Apis* species are more likely the result of neutral evolution [51].

Honey bee colonies can use several behavioral strategies to avoid the reciprocal intrusion of foreign individuals. Drifting, where a forager visits a foreign colony instead of the mother colony, is particularly likely in nest aggregations. To minimise drifting, foragers must be able to identify the position of the mother colony via landmarks even in a dense nest aggregation. A potential colony-specific nest odour could be quite helpful as a strategy to confirm the identification of their own nest via landmarks. In addition, colonies can effectively prevent the intrusion of non-nestmates through their guards. Considering the behavior of drifters, we observed that in *A*. *dorsata* non-nestmates were less motivated to approach a foreign conspecific colony than *A*. *florea* non-nestmates, suggesting that in *A*. *dorsata* homing workers are more cautious when approaching a colony. Considering guarding behavior, we found that both *A*. *dorsata* and *A*. *florea* workers were able to recognize and repel non-nestmates. This finding is in contrast to an earlier study that used a lab assay with individual workers and could not show non-nestmate discrimination in *A*. *dorsata* and *A*. *florea* [52]. Our CHC dummy experiments using colonies of *A*. *dorsata and A*. *florea* under natural conditions showed that this two honey bee species could use CHCs profiles for nestmate discrimination. Furthermore, in accordance with our hypothesis, *A*. *dorsata* workers rejected intruding conspecific non-nestmates at a much higher rate than *A*. *florea* workers. Our findings are supported by Weihmann et al. [14] who also reported that *A*. *dorsata* colonies fend off intruding foreign workers under natural conditions. This study showed that *A*. *dorsata* colonies are capable of detecting non-nestmates by aberrant flight approaches and respond to this with shimmering behavior. We did not record shimmering responses in our experiments. However, both findings do not contradict each other, instead suggest different stages of defensive responses, the first, a long-range response based on visual cues, and the second, a short-range response based on olfactory cues. In this case, increased information content from methyl-branched alkanes may be beneficial. Alternatively, larger amounts of methyl-branched alkanes could help counteract possible desiccation stress in *A*. *dorsata* individuals hanging exposed on tall trees [53].

The evolution of a more fine-tuned nestmate recognition ability at close range in *A*. *dorsata* may represent an adaptation for nesting in close aggregations which is unique among honey bee species [8]. Naturally, nests of *A*. *mellifera* are widely spaced in the environment so that drifting events are unlikely to occur [1, 54]. In apiaries however, beekeepers artificially aggregate colonies of these species by placing hives next to each other. It is well documented that under such unnatural conditions drifting of foragers between nests is common. Up to 40% of drifted bees can be found in artificially aggregated colonies in apiaries [55]. The extremely high tolerance towards non-nestmates observed in apiaries with *A*. *mellifera* colonies might be due to guard bees which lack the ability to discriminate non-nestmates from nestmates using olfactory cues. The lack of fine-tuned nestmate discrimination is not as important in species such as *A*. *mellifera*, which under natural conditions singularise their nests in the field and arrange them further apart from each other. On the contrary, the ability to distinguish foreign bees from nestmates, especially at short distances, allows a dense arrangement of colonies while maintaining colony integrity, which, as observed in nest aggregations of *A*. *dorsata*, are even arranged side by side on thick tree branches or on rocky niches.

## Conclusions

In our study, we show that foragers of the giant honey bee *A*. *dorsata* have a complex cuticular hydrocarbon profile compared to other honey bee species, such as *A*. *mellifera*, *A*. *cerana* and *A*. *florea*. This complex profile might provide a better distinguishable olfactory signature to identify non-nestmates. These findings are in congruence with the nesting ecology of *A*. *dorsata*, whose colonies aggregate in large numbers on single trees often only a few centimeters away from each other. Thus, it allows *A*. *dorsata* to maintain their social integrity to keep non-nestmates efficiently out of their colony which is in line with studies using behavioral and molecular markers [12, 13]. Although, we have evidence that *A*. *dorsata* uses chemical cues from the cuticle to discriminate between nestmates and non-nestmates, we are aware that this honey bee species also uses visual cues for nestmate recognition [14].

There are two possible evolutionary scenarios to explain the present CHC phenotypes: First, methyl-branched alkanes in the forager stage represent the ancestral profile and *A*. *florea*, *A*. *mellifera*, and *A*. *cerana* experienced an evolutionary loss. Second, and this might be the more parsimonious scenario, the ancestral forager phenotype expressed only alkanes and unsaturated hydrocarbons as is found in *A*. *florea* and the other two investigated honey bee species. In this case, *A*. *dorsata* might have reactivated the biosynthesis of methyl-branched alkanes, which was present in the pupal stage, in the adult stage to establish a more sensitive nestmate recognition system as a consequence of nesting in close aggregation with conspecific colonies. The results of our study suggest that the species-specific behavioural trait of nesting in aggregations also results in a species-specific diversification of the CHC profile.

## Supporting information

S1 Table Summary of post hoc-test statistics. (XLSX)

S2 Table Raw data GCMS. (CSV)

S3 Table Raw data colony response against cuticular hydrocarbon odor. (CSV)

S4 Table Raw data colony response on intruding attempts. (CSV)

S1 File Colony specificity of CHC profiles. (PDF)
