# Supplementary material for: Increased complexity of worker CHC profiles in Apis dorsata correlates with nesting ecology
Source: PLoS One. 2022 Jul 28;17(7):e0271745. doi: 10.1371/journal.pone.0271745 (PMC9333238; doi:10.1371/journal.pone.0271745)
Supplement: S1 File — (PDF) [file pone.0271745.s005.pdf]

S5 Figure. Colony specificity of CHC profiles

We addressed colony specificity of CHC profiles by analyzing the relative abundances of compounds of three colonies per species. We used Bray-Curtis distances as measures of profile similarity. We revealed profile composition differences between colonies of the same species by performing pairwise permutational multivariate analysis of variance (PERMANOVA) and Non-metric Multidimensional Scaling (NMDS).

Please note, we interpret the different cluster as colony specific profiles. For methodological similar identified colony specificity in *A. mellifera* see (Vernier et al., 2019)

1. Colony specificity of CHC profiles in *Apis dorsata*

Colonies in *A. dorsata* differ significantly (see Table 1 below).

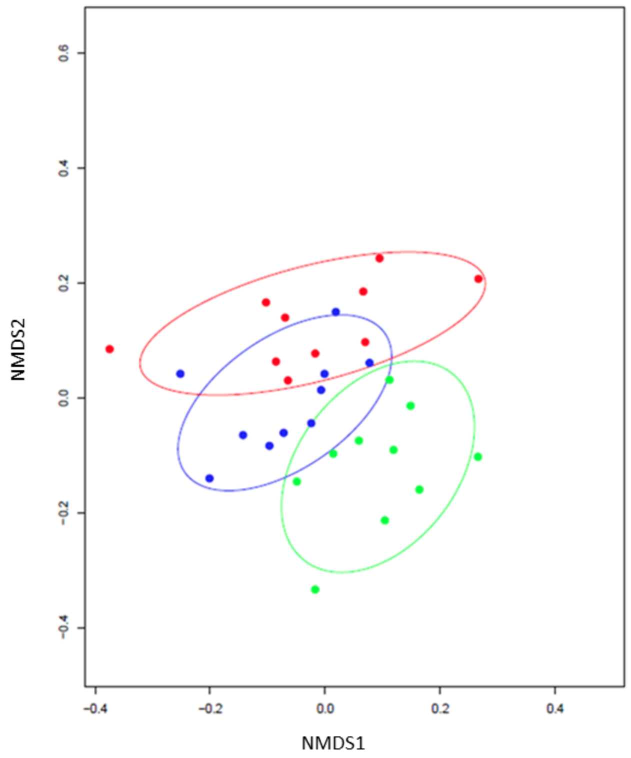

Figure 1 NMDS of Apis dorsata colonies. Each color represents a colony (red= colony1; blue= colony2, green= colony3)

Table 1 Pairwise colony comparison: given are p-values and Bonferroni corrected p-values

pairs	p-value	p-value Bonferroni corrected
colony1 vs colony2	0.0002	0.0006
colony1 vs colony3	<0.0001	0.0002
colony3 vs colony2	0.005	0.0151

2. Colony specificity of CHC profiles in *Apis florea*

Colonies in *A. florea* differ significantly (see Table 2 below).

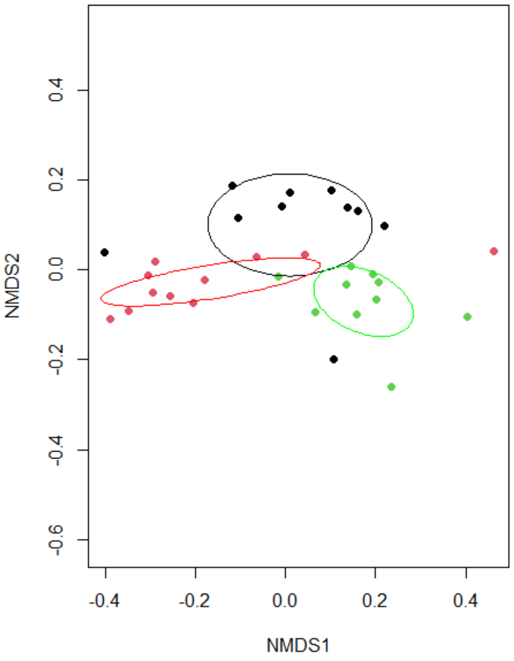

Figure 2 NMDS Apis florea colonies. Each color represents a colony (red= colony1; black= colony2, green= colony3)

Table 2 Pairwise colony comparison: given are p-values and Bonferroni corrected p-values

pairs	p-value	p-value Bonferroni corrected
colony1 vs colony2	0.008	0.024
colony1 vs colony3	0.001	0.003
colony3 vs colony2	0.001	0.003

References

Vernier, C. L., Krupp, J. J., Marcus, K., Hefetz, A., Levine, J. D., & Ben-Shahar, Y. (2019). The cuticular hydrocarbon profiles of honey bee workers develop via a socially-modulated innate process. *eLife*, *8*, e41855. https://doi.org/10.7554/eLife.41855
